# Assessment of Physicochemical Characterization and Mineralization of Nanofibrous Scaffold Incorporated With Aspartic Acid for Dental Mineralization: An In Vitro Study

**DOI:** 10.7759/cureus.61741

**Published:** 2024-06-05

**Authors:** Aruna Krishnan, Sandhya Raghu, Priyadharsan Arumugam, Rajalakshmanan Eswaramoorthy

**Affiliations:** 1 Department of Conservative Dentistry and Endodontics, Saveetha Dental College and Hospital, Saveetha Institute of Medical and Technical Sciences, Saveetha University, Chennai, IND; 2 Department of Cariology, Saveetha Dental College and Hospital, Saveetha Institute of Medical and Technical Sciences, Saveetha University, Chennai, IND; 3 Department of Biochemistry, Center of Molecular Medicine and Diagnostics (COMManD), Saveetha Dental College and Hospital, Saveetha Institute of Medical and Technical Sciences, Saveetha University, Chennai, IND

**Keywords:** noncollagenous proteins, scaffold, dentin mineralization, polycaprolactone, nanofibers, aspartic acid

## Abstract

Aim

The aim of this study was to assess the physicochemical characterization and mineralization of nanofibrous scaffold incorporated with nanohydroxyapatite (nHA) and aspartic acid (Asp) for dental mineralization.

Methodology

Three nanofibrous scaffolds were prepared, namely polycaprolactone (PCL), PCL with nHA, and PCL with nHA and Asp. Each scaffold was prepared separately by electrospinning. The physicochemical characterization of the surface of the nanofibrous scaffold was imaged using a scanning electron microscope (SEM), energy dispersive X-ray Analysis (EDX), X-ray diffraction (XRD), and Fourier-transform infrared spectroscopy (FTIR). In vitro mineralization studies were performed by immersing the sample in simulated body fluid (SBF) for 7, 14, and 21 days. The surface of the samples was observed under SEM with EDX.

Results

SEM analysis of PCL/nHA/Asp revealed that the nanoﬁbers were bead-free, smooth, randomly oriented, and loaded with Asp. The EDX spectra of PCL/nHA/Asp composite nanofibrous scaffold revealed broad peaks and corresponded to the amorphous form, while the sharp peaks corresponded to the specific crystalline structure of nHA. FTIR analysis showed specific functional groups corresponding to PCL, nHA, and Asp. The scaﬀolds incorporated with Asp exhibited higher mineralization potential with an apatite-like crystal formation, which increased with an increase in the duration of immersion in SBF.

Conclusion

Physiochemical characterization demonstrated the incorporation of PCL/nHA/Asp in the electrospun nanofibrous scaffold. The mineralization analysis revealed that the presence of Asp enhanced the mineralization when compared with the PCL and PCL/nHA. PCL/nHA/Asp incorporated in scaffold can be a promising material for dental mineralization.

## Introduction

Dentin, a biomineralized hard tissue, comprises approximately 70% hydroxyapatite (HA), around 20% organic matrix mainly composed of type I collagen fibrils, and roughly 10% water [1]. Enamel primarily comprises inorganic material (96%), with a small proportion of organic substance and water comprising only 4% of its composition [1]. Unlike enamel remineralization, dentin remineralization is complicated. The process of mineralizing hard connective tissues such as bone and dentin is complicated, encompassing HA's incorporation into a collagen-based structure [2]. Dental caries is a biofilm-mediated, diet-modulated, multifactorial, non-communicable, dynamic disease resulting in net mineral loss of dental hard tissues [3]. Throughout a lifetime, teeth, being highly mineralized, undergo continual cycles of demineralization and remineralization. Dental caries represents a dynamic disease process resulting from an imbalance between the demineralization and remineralization of dental hard tissues [4]. Caries process in dentin happens in two stages. Examinations at an ultrastructural level have indicated that demineralization and the degradation of the organic matrix occur in two consecutive stages [5,6]. During the initial phases of cementum and dentin caries, minerals are dissolved in a gradual manner starting from the outer surface. Yet, the distinctive cross-banding pattern of the collagen fibers remains intact [5]. Demineralized collagen acts as a framework for bacteria to colonize. As the condition progresses, the exposed collagen undergoes degradation due to proteolytic enzymes, causing the collagen fibers to lose their structural features. Understanding how minerals are deposited onto collagen is crucial for advancing treatments aimed at mineralization-related diseases. Moreover, this understanding holds significant value in crafting bioinspired materials to repair hard tissues. Both collagen and noncollagenous proteins (NCPs) play roles in regulating the initiation, nucleation, growth, and prevention of HA formation during the creation of hard tissues [7]. It is acknowledged that decayed dentin comprises two separate layers characterized by distinct ultramicroscopic and chemical compositions. The outer layer, known as infected dentin, undergoes irreversible denaturation, is infected, and cannot be remineralized; therefore, it needs to be eliminated. In contrast, the inner layer, termed affected dentin, experiences reversible denaturation, lacks infection, and can be remineralized, thus requiring preservation [8].

Hence, the remineralization of non-infected carious dentin holds importance in preserving tooth tissues during vital pulp therapy, aligning with the principles of minimally invasive dentistry. The restoration of dentin mineralization is crucial to the prevention and treatment of dental caries, as well as the maintenance of long-term oral health. Remineralization of dental tissue involves restoring the lost mineral fraction after the demineralization process. Clinically, effective remineralization of a demineralized dentin layer would be beneficial in preventing or treating conditions such as dentin caries, root caries, and dentin hypersensitivity. Implementing these strategies forms the basis for minimally invasive methods in managing the initial stages of dental caries. The process of remineralization occurs through the epitaxial growth of existing mineral crystals within demineralized lesions under favorable conditions. Hence, the effectiveness of remineralization significantly hinges on the presence of these residual crystals, forming the inorganic matrix [9]. Currently, the widely accepted belief is that the process of dentin remineralization does not occur through spontaneous mineral precipitation or the nucleation of minerals on the organic matrix, primarily composed of type I collagen. Instead, it is believed to result from the growth of existing inorganic crystals within the lesions [10]. In the process of biomineralization of dentin, the collagen matrix can serve as a framework for mineral deposition in the presence of NCPs such as dentin matrix protein 1 (DMP1) and dentin phosphophoryn (DPP, DMP2), which contain highly phosphorylated serine and threonine residues [11]. These NCPs play a role in initiating and controlling dentin biomineralization in vivo by acting as either nucleators or inhibitors. Leveraging the understanding of the nucleating function of NCPs in dentin biomineralization, it is plausible to replicate this natural mechanism to promote remineralization of demineralized dentin. Modifying dentin collagen to induce mineralization could enhance the remineralization of demineralized dentin. The functional groups present in NCPs are believed to serve as sites for mineralization. Among these groups, phosphate, carboxyl, amido, and hydroxyl exhibit a descending order of binding affinity to calcium ions [11,12]. Hence, directly incorporating specific functional groups of NCPs into collagen might be a viable approach to stimulate collagen mineralization.

The study aimed to develop a scaffold with polycaprolactone (PCL), nanohydroxyapatite (nHA), and aspartic acid (Asp) using the electrospinning technique. The developed nanofibrous scaffold was then characterized using a scanning electron microscope (SEM), energy-dispersive X-ray spectroscopy (EDX), X-ray diffraction (XRD), and Fourier-transform infrared spectroscopy (FTIR). The mineralization study was conducted by immersing the scaffold in simulated body fluid (SBF) and then imaged using SEM-EDX.

## Materials and methods

Materials

PCL pellets (Mw: 73,000-80,000), nHA powder 200 nm, and Pluronic F127 were procured from Sigma-Aldrich (Bangalore, India). Asp was obtained from Southern India Scientific Corporation (Chennai, India). Dimethylformamide (DMF), chloroform (CHCl3), minimum essential medium (MEM), and 1% antibacterial-antifungal solution of 100× were purchased from HiMedia Laboratories (Thane, India).

Fabrication of nanofibrous scaffolds by electrospinning

**Figure 1:**
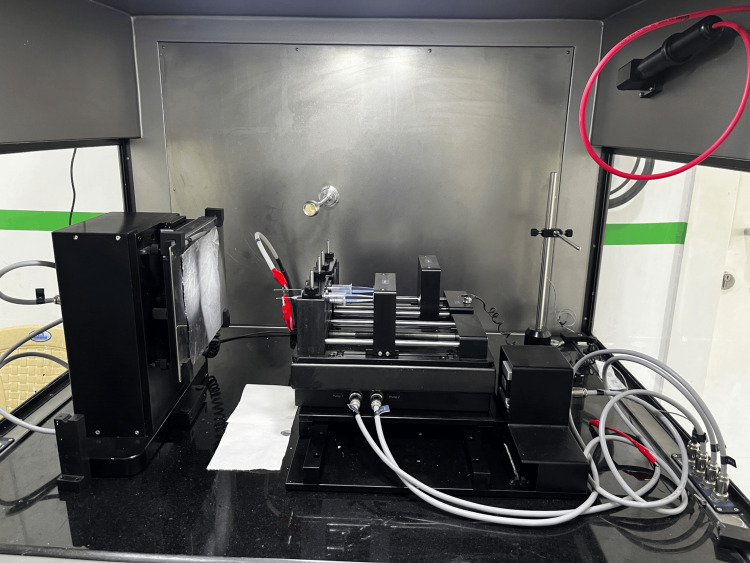
Fabrication of nanofibrous scaffold using the electrospinning method

Three nanofibrous scaffolds prepared using the electrospinning method were PCL, PCL/nHA, and PCL/nHA/Asp (Figure. 1). The prescribed amount of PCL pellets was dissolved in a mixture of DMF and CHCl3, creating a polymer solution of 10 wt%. Similarly, a solution containing PCL and nHA was prepared. The third preparation contained PCL with 2 wt% Asp soluble in DMF and nHA. Continuous stirring overnight at room temperature ensured homogeneity. The solutions were then loaded into syringes for electrospinning. An aluminum sheet foil on the collector plate, connected to the negative electrode, facilitated the deposition of electrospun fibers. Parameters including flow rate (500 μL/hour), applied voltage (10-15 kV), and needle tip to collector distance (7.5 cm) were consistent across all solutions. Electrospinning continued for 3 hours to form mats. Subsequently, samples were placed in a vacuum desiccator overnight to remove residual solvent. The resulting fibers were stored securely for further analysis. Samples were labeled as PCL, PCL/nHA, PCL/nHA/Asp.

Physicochemical characterization

The samples' surfaces underwent gold sputter coating and were then examined using an SEM with EDX (JSM IT800, JEOL Ltd., Tokyo, Japan). The mean fiber diameter was assessed by randomly choosing 100 fibers through Image J software. For the analysis of functional groups in both the starting materials and the produced nanofibers, FTIR spectroscopy was carried out using a JASCO 4100 spectrometer (Bruker, Billerica, MA, USA). Spectral assessments spanned from 400 to 4,000 cm^−1^, with each sample subjected to 32 scans. The crystallinity of the samples was analyzed using an X-ray diffractometer (Bruker, Biopolis St, Singapore) at a step size of 0.1° and a scanning speed of 1 step/s.

In vitro biomineralization study in SBF

The ability of polymer scaffolds to generate apatite can be assessed by placing them in conditions that mimic the body conditions, such as SBF in a laboratory setting. SBF replicates the ion concentrations found in human blood plasma, providing a near-physiological environment for evaluation. The electrospun PCL, PCL/nHA, PCL/nHA/Asp samples (three replicates) were cut out into dimensions of 60±10 mm x 60±10 mm. The pH of the SBF solution was regulated to 7.4 ± 0.1 at a temperature of 36.5 ± 0.2 degrees Celsius. The samples were then placed in SBF and incubated at 36.5 degrees Celsius for three time periods: 7 days, 14 days, 21 days. Ultimately, the samples underwent drying within a desiccator at room temperature for 48 hours. The samples were then imaged using SEM with EDX.

Statistical analysis 

The calcium and phosphate wt% were assessed using EDX at baseline, 7 days, 14 days, and 21 days across three different groups: PCL, PCL/nHA, and PCL/nHA/Asp. Statistical analysis was performed using one-way ANOVA followed by a post-hoc Tukey HSD test. Differences were considered significant when P-value was < 0.05.

## Results

SEM-EDX analysis of the scaffold

**Figure 2:**
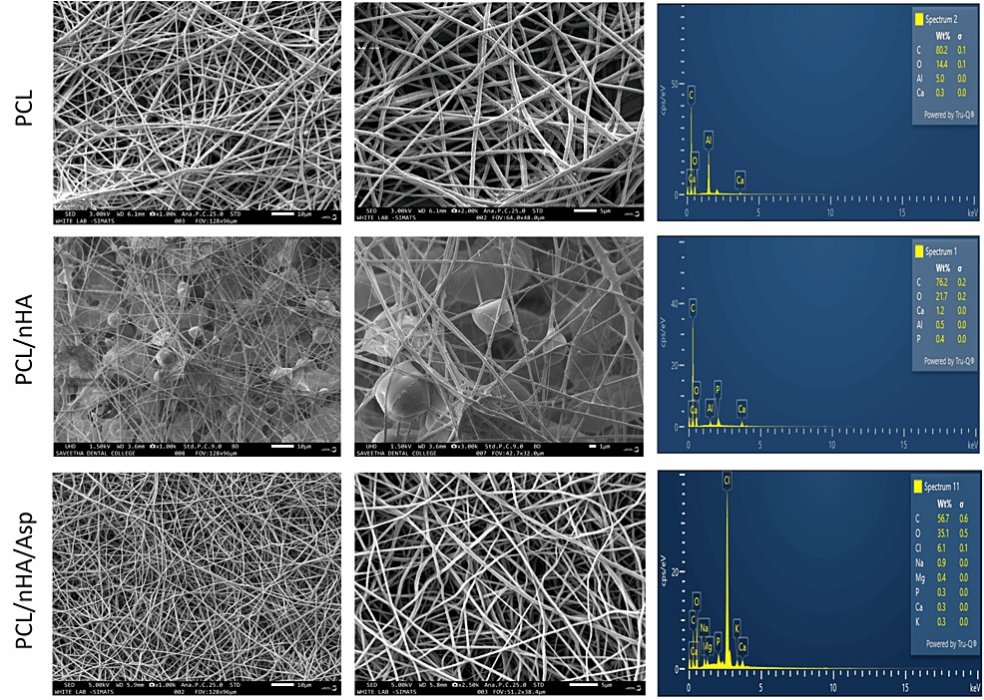
SEM images of electrospun nanofibrous scaffold PCL, PCL/nHA, and PCL/nHA/Asp at two different magnifications and their EDX spectrum C, carbon; O, oxygen; Cl, chlorine; Na, sodium; Mg, magnesium; P, phosphorus; Ca, calcium; K, potassium; SEM, scanning electron microscope; PCL, polycaprolactone; nHA, nanohydroxyapatite; Asp, aspartic acid; EDX, energy dispersive X-ray analysis

Electrospun PCL scaffolds were successfully produced with various formulations. SEM images showed that the nanofibrous scaffolds PCL, PCL/nHA, PCL/nHA/Asp were bead-free, smooth, and uniform (in Figure 2). Figure 2 represents SEM images detailing the morphological features of 10% PCL nanofibers at 1,000x and 2,000x magnifications, indicating an average diameter of approximately 600 nm. Each formulation yielded fiber meshes that were distributed randomly. The diameter of the PCL/nHA/Asp nanofibers was higher when compared to PCL/nHA and PCL scaffolds. This property is due to the addition of Asp and nHA, which increased the viscosity of the polymer solution leading to increased fiber diameter. Likewise, the PCL/nHA scaffold with the addition of pluronic showed decreased fiber diameter. This property is due to the addition of this hydrophilic polymer, which increased the homogeneity of the polymer solution; hence, a decrease in fiber diameter was observed. Interconnected fibers exhibit noticeable randomness attributed to bending instability during electrospinning. This randomness underscores the dynamic nature of fabrication, leading to morphological transformations. EDX was used to verify the existence of nHA within the PCL polymer solution. EDX confirms the presence of calcium and phosphate corresponding to nHA in PCL/nHA and PCL/nHA/Asp scaffolds.

Additionally, EDX analysis unveiled the elemental composition of PCL nanofibers, with carbon constituting 80%, oxygen at 14%, and minor traces of aluminum and calcium, collectively comprising the remaining 6%. The SEM images (Figure 2, PCL/nHA) exhibited a well-dispersed network with intermittent bead formations due to variations in polymer viscosity during electrospinning, indicative of the homogenous incorporation of both materials. The accompanying (EDX) analysis further elucidated the elemental composition. Carbon and oxygen constituted 76% and 22%, respectively, reflecting the PCL matrix. The presence of calcium, aluminum, and phosphate in the remaining percentage aligns with the expected composition of nHA. The SEM analysis of the nanofibers comprising PCL, nHA, and Asp (Figure 2) reveals a uniform and continuous fibrous structure without discernible bead formations. This absence of bead formations suggests a consistent and controlled electrospinning process, emphasizing the precision in the fabrication of the nanofibrous scaffold. The (EDX) analysis provides insights into the elemental composition. Carbon and oxygen constitute 56% and 35%, respectively, reflecting the PCL and Asp components. The presence of chlorine, sodium, phosphorus, magnesium, calcium, and potassium in the remaining composition aligns with the anticipated elements of nHA and Asp.

XRD analysis and FTIR analysis

**Figure 3:**
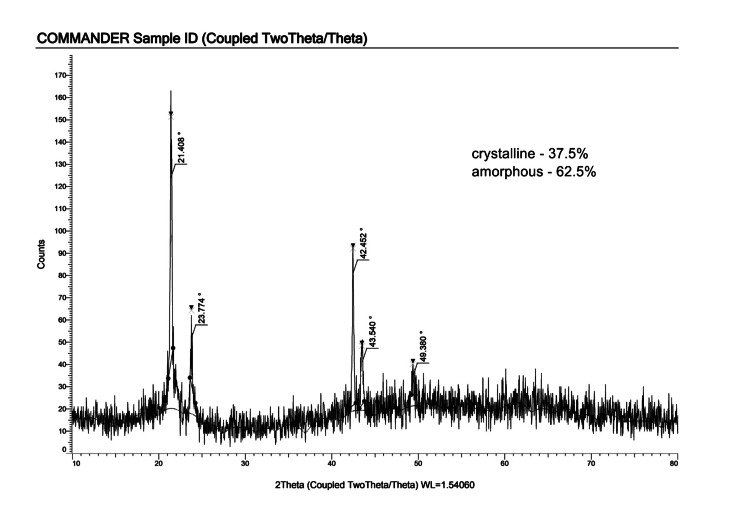
XRD image of PCL/nHA/Asp scaffold XRD, X-ray diffraction analysis; PCL, polycaprolactone; nHA, nanohydroxyapatite; Asp, aspartic acid

XRD analysis showed the characteristic peak corresponding to the individual component and also showed crystalline peaks for the Asp (Figure 3). In Figure 3, the diffraction patterns of the prepared composite, comprising PCL, nHA, and Asp, reveal distinctive peaks at specific angles corresponding to crystallographic planes of nHA. These findings confirm the crystalline nature of nHA within the composite, with the (020) and (112) planes showing significant prominence, as indicated by both net and gross intensity values. While the XRD results primarily highlight the crystallinity of nHA, the inclusion of Asp introduces the possibility of its role in influencing the composite's properties.

**Figure 4:**
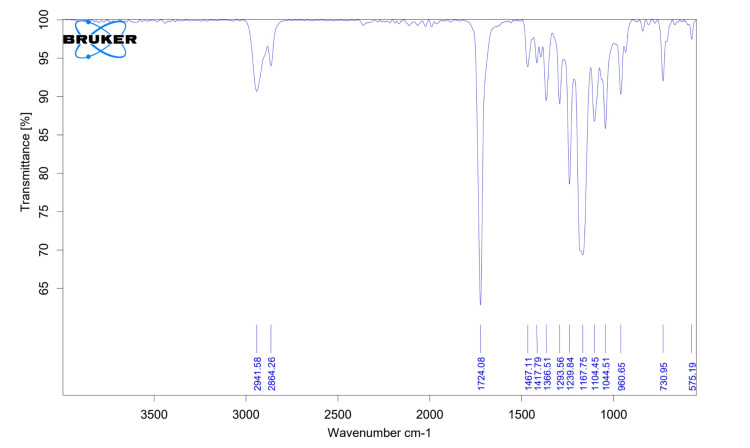
FTIR spectrum of PCL/nHA/Asp scaffold FTIR, Fourier-transform infrared spectroscopy; PCL, polycaprolactone; nHA, nanohydroxyapatite; Asp, aspartic acid

FTIR analysis showed characteristic functional groups corresponding to carbonyl stretching of PCL, OH peak of nHA, and NH2 peaks corresponding to Asp (Figure 4). The spectrum of the composite, comprising PCL, nHA, and Asp, represented in Figure 4 manifests distinctive peaks indicative of various functional groups. The peaks observed at 2,941.58 cm⁻¹ and 2,864.26 cm⁻¹ are attributed to C-H stretching within the aliphatic structure of PCL, thereby confirming its molecular presence. The peak observed at 1,724.08 cm⁻¹ denotes the carbonyl stretching vibration in PCL, providing affirmative evidence for the existence of the ester group within the composite. Peaks at 1,366.51 cm⁻¹, 1,239.84 cm⁻¹, and 1,167.75 cm⁻¹ collectively suggest the successful integration of nHA, reflecting the presence of phosphate and carbonate groups. Significantly, the peak observed at 1,293.56 cm⁻¹ signifies the incorporation of Asp into the composite, emphasizing the multifunctional nature of this material.

In vitro biomineralization study in SBF

**Figure 5:**
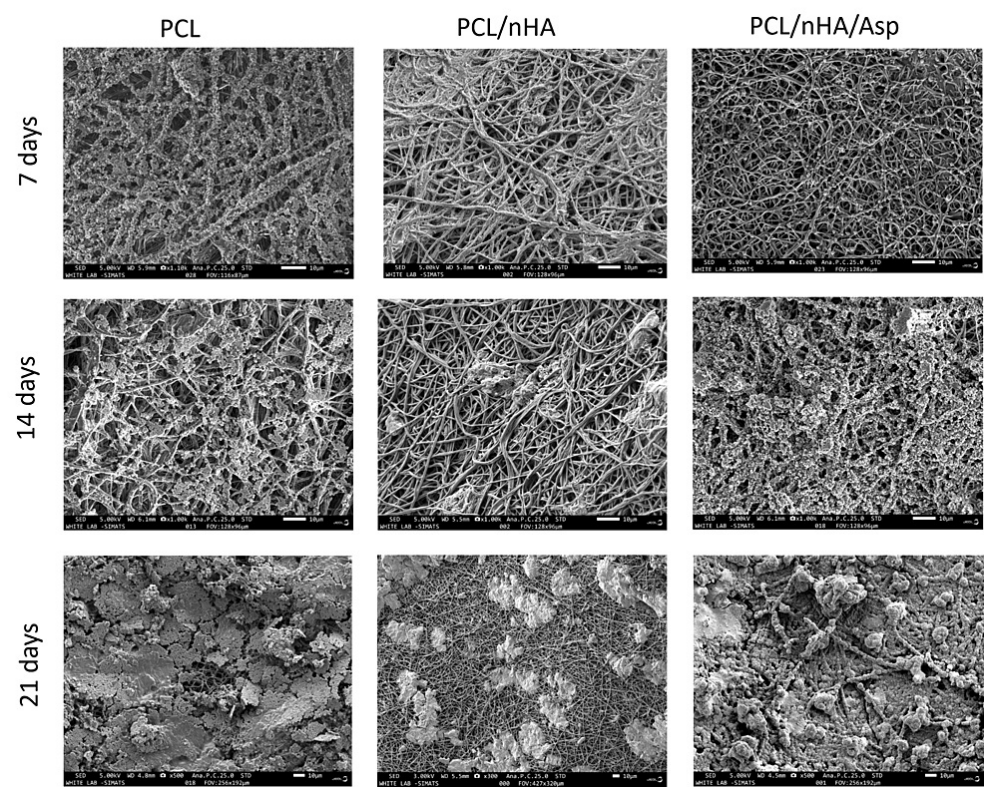
Representative SEM images of three nanofibrous scaffolds PCL, PCL/nHA, and PCL/nHA/Asp following immersion in SBF After 7 days of incubation, in PCL scaffold, tiny globular-shaped crystals were observed on the surface of the fiber and not within the pore network. In PCL/nHA scaffold, dense deposits in between the nanofibers were observed. In PCL/nHA/Asp scaffold, small spherical seeding crystals were observed along the fibers, serving as the initial points for crystal growth to occur. After 14 days of incubation, the PCL scaffold showed granular deposits only along the surface of the nanofiber. PCL/nHA scaffold showed randomly oriented mineral deposition. PCL/nHA/Asp scaffold produced cauliflower-shaped globular deposits along the nanofibers, indicating crystal growth. After 21 days of incubation, PCL showed intensive deposition of minerals only on the surface of the fiber and not within the pore network. PCL/nHA scaffold showed unevenly distributed intensive mineral deposits. PCL/nHA/Asp scaffold showing the distribution of crystals formed all over the fibers. Crystal growth was seen along a uniform axis, producing uniform deposits all over the fibers. SEM, scanning electron microscope; PCL, polycaprolactone; nHA, nanohydroxyapatite; Asp, aspartic acid; SBF, simulated body fluid

**Figure 6:**
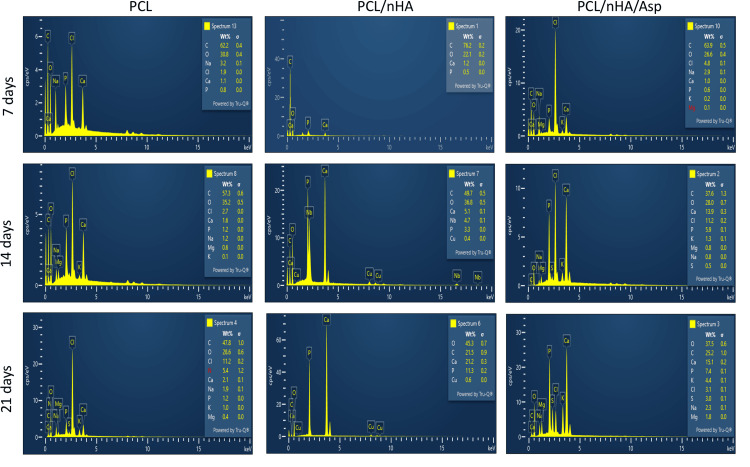
EDX on the surface of PCL, PCL/nHA, and PCL/nHA/Asp following placement in the SBF for 7, 14, and 21 days C, carbon; O, oxygen; Cl, chlorine; S, sulfur; Ca, calcium; Na, sodium; P, phosphorus; K, potassium; Mg, magnesium; EDX, energy-dispersive X-ray spectroscopy; PCL, polycaprolactone; nHA, nanohydroxyapatite; Asp, aspartic acid; SBF, simulated body fluid

Representative SEM micrographs of scaffold samples incubated in SBF for 7, 14, and 21 days’ duration are shown in Figure 5. After 7 days of incubation, in PCL scaffold, tiny globular-shaped crystals were observed on the surface of the fiber and not within the pore network. In PCL/nHA scaffold, dense deposits in between the nanofibers were observed. In PCL/nHA/Asp scaffold, increase in the width of the fibers was observed due to the uniform deposition of minerals along the fibers. Small spherical seeding crystals were observed along the fibers, serving as the initial points for crystal growth to occur. Increasing the incubation time in SBF from 7 days to 14 days led to substantial crystal nucleation and growth. Apatite crystals that developed on the nanofibrous scaffold loaded with Asp and within the nearby pore structure displayed a rounded, cauliflower-like appearance, resembling clusters of HA crystals [13,14]. It can be hypothesized that an initial calcium phosphate layer is created on the surface of the fibers within the scaffold. Subsequently, additional growth of spherical clusters takes place over this layer. In vitro biomineralization study showed that increased mineralization was observed for Asp-incorporated scaffolds for the duration of 14 days. This improved mineralization is due to the addition of nHA and Asp as a biomimetic analogue of NCPs, the hydrophilic nature of the scaffolds, and PCL, which served as an ECM. The increase in the amount of crystal deposits on the scaffolds with increased incubation time was assessed by EDX (Figure 6).

**Figure 7:**
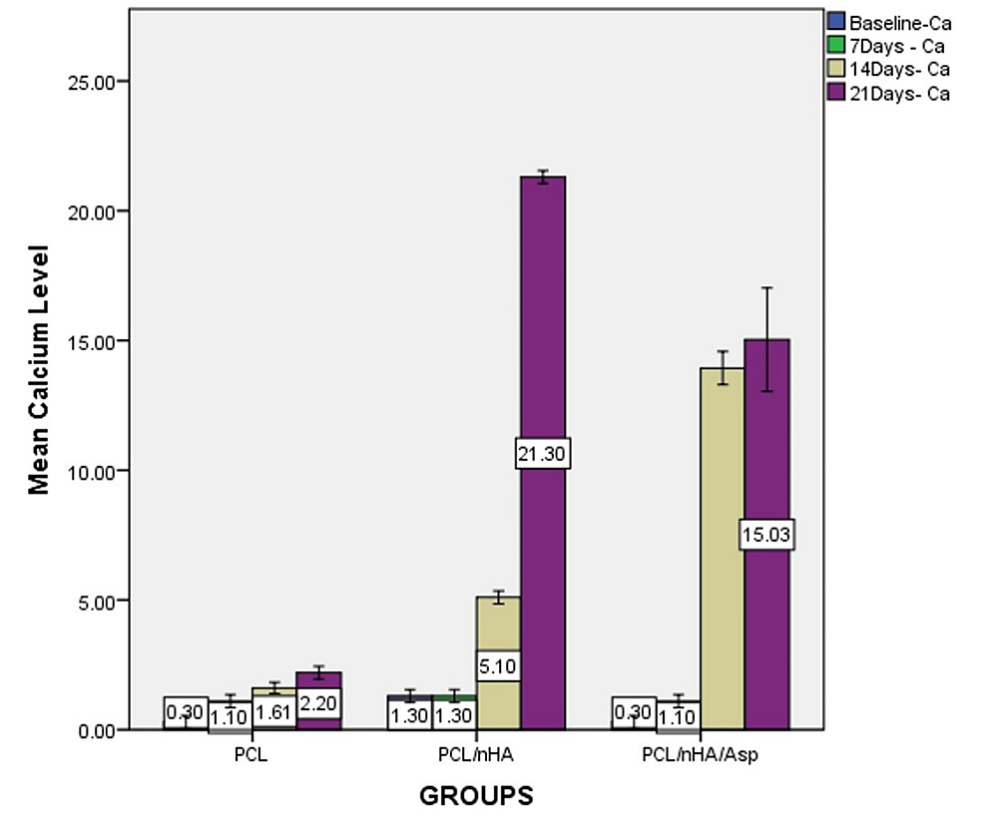
Graph showing calcium wt% assessed using EDX on the surface of PCL, PCL/nHA, PCL/nHA/Asp immersed in SBF for 7, 14, and 21 days EDX, energy-dispersive X-ray spectroscopy; PCL, polycaprolactone; nHA, nanohydroxyapatite; Asp, aspartic acid; SBF, simulated body fluid

**Figure 8:**
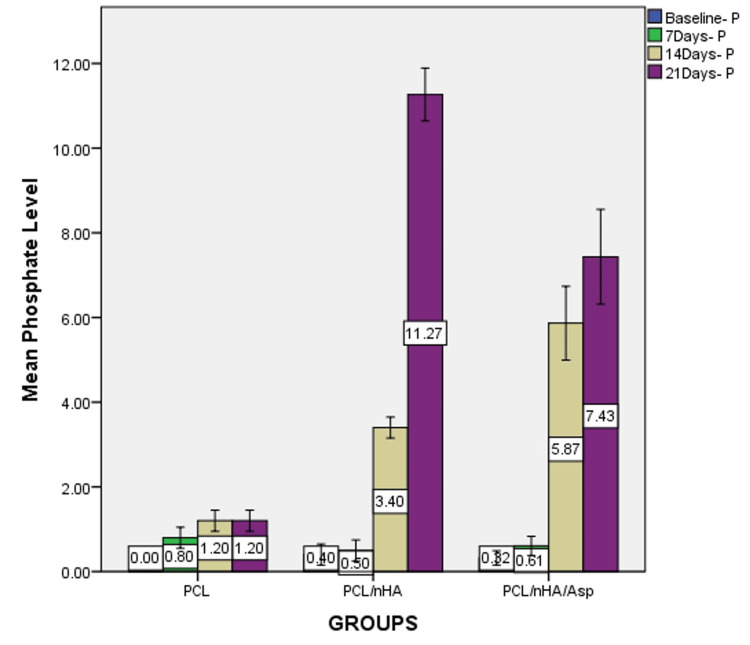
Graph showing phosphate wt% assessed using EDX on the surface of PCL, PCL/nHA, PCL/nHA/Asp immersed in SBF for 7, 14, and 21 days EDX, energy-dispersive X-ray spectroscopy; PCL, polycaprolactone; nHA, nanohydroxyapatite; Asp, aspartic acid; SBF, simulated body fluid

Based on the one-way ANOVA, at 7 days, calcium wt% did not show significant difference between the groups (P = 0.079) but showed a significant difference in phosphate levels between the groups (P=0.024). At 14 and 21 days, significant difference was observed in the calcium and phosphate levels between the groups (P = 0.000). Post-hoc analysis revealed significant differences between all the groups (Figures 7, 8).

**Figure 9:**
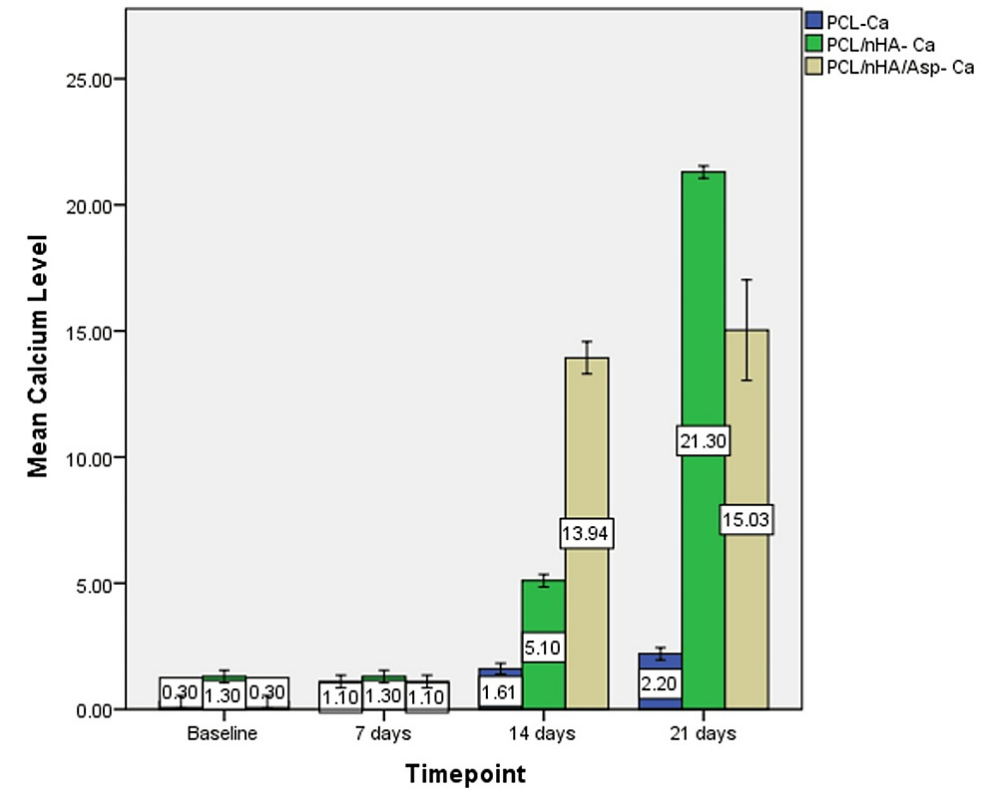
Graph showing calcium wt% at baseline, 7, 14, and 21 days of immersion in SBF assessed using EDX on the surface of PCL, PCL/nHA, and PCL/nHA/Asp EDX, energy-dispersive X-ray spectroscopy; PCL, polycaprolactone; nHA, nanohydroxyapatite; Asp, aspartic acid; SBF, simulated body fluid

At 7 days, calcium level was highest in PCL/nHA. At 14 days, the calcium level was highest in PCL/nHA/Asp followed by PCL/nHA and PCL. At 21 days, the calcium level was highest in PCL/nHA followed by PCL/nHA/Asp and PCL (Figure 9).

**Figure 10:**
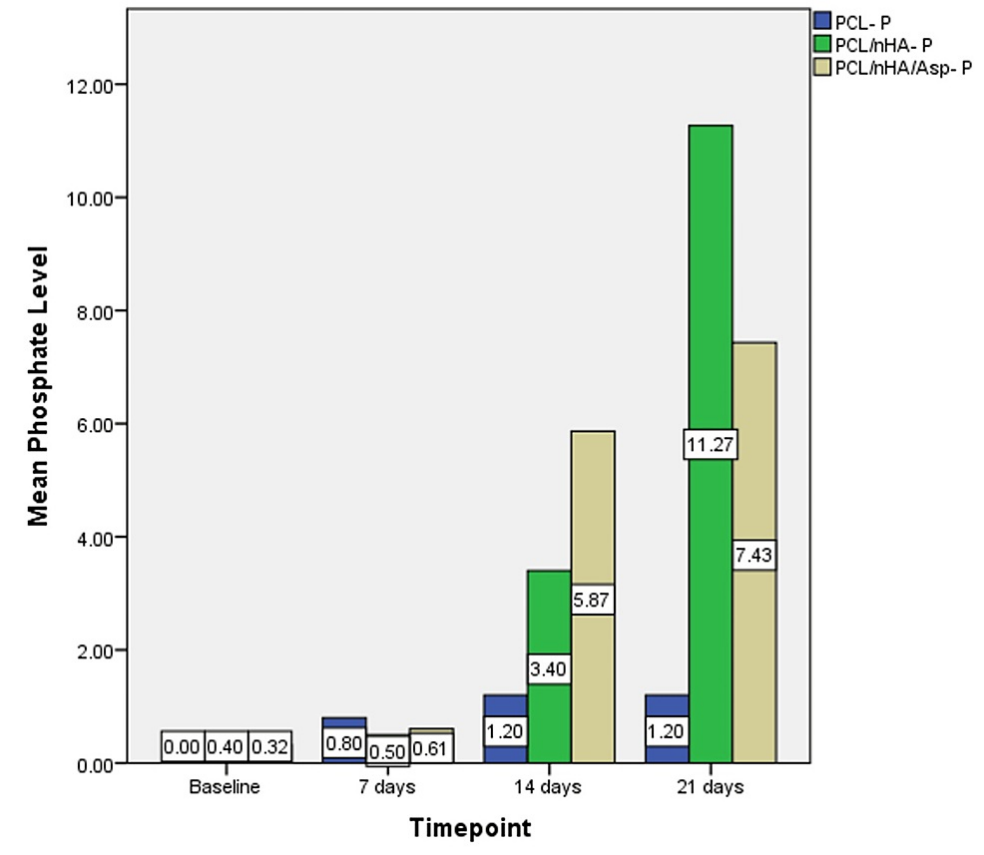
Graph showing phosphate wt% at baseline, 7, 14, and 21 days of immersion in SBF assessed using EDX on the surface of PCL, PCL/nHA, and PCL/nHA/Asp EDX, energy-dispersive X-ray spectroscopy; PCL, polycaprolactone; nHA, nanohydroxyapatite; Asp, aspartic acid; SBF, simulated body fluid

At 7 days, the phosphate level was highest in PCL followed by PCL/nHA/Asp and PCL/nHA. At 14 days, the phosphate level was highest in PCL/nHA/Asp followed by PCL/nHA and PCL. At 21 days, the phosphate level was highest in PCL/nHA followed by PCL/nHA/Asp and PCL (Figure 10).

PCL/nHA/Asp showed higher calcium and phosphate levels compared to PCL/nHA at 14 days (P = 0.000) and lower levels at 21 days (P = 0.000).

## Discussion

The dentin matrix contains numerous NCPs. Among these, dentin phosphoprotein (DPP) and dentin sialoprotein (DSP) are notably prevalent. They represent cleavage fragments of DSPP post-protein hydrolysis, with DSP at the amino terminus and DPP at the carboxyl terminus. DPP stands as the most prevalent protein among NCPs, making up approximately 50% of this category [15]. DPP is rich in Asp and phosphorylated serine, commonly located within repeat sequences such as (Asp-Pse-Pse)n and (Asp-Pse)n [16]. These proteins interact with the positively charged calcium ions, facilitating their organized arrangement and actively participating in both nucleation and regulation during the formation of apatite crystals [17]. DMP1 is an NCP found abundantly in dentin. It primarily consists of Asp, glutamic acid, and serine. Due to the high presence of carboxyl structures within these amino acids, DMP1 appears highly acidic and extensively phosphorylated as a NCP [17,18]. It carries a significant negative charge, enabling strong interactions with calcium ions, thereby actively engaging in the mineralization process of dentin.

Composite materials in the form of scaffolds have been developed. They replicate the collagen matrix and the mineralized aspect of the ECM. It would function as a framework for regenerating mineralized tissue [19]. The nanofibrous scaffolds have unique properties such as high surface area, interconnected porosity, and resemblance to the ECM architecture [20]. These properties are crucial for tissue regeneration characteristics. Commonly used biocomposite configurations for bone regeneration typically include a biodegradable polymer scaffold, mimicking the structure or function of collagen, alongside an inorganic phase such as synthetic HA. This choice is based on the biocompatibility, absence of toxicity, and osteoinductive properties of HA [21,22].

PCL, an FDA-approved biodegradable polymer, has found to be extensively used alongside HA in applications related to bone tissue engineering [23,24]. Biodegradable polymer scaffolds are mostly used for bone regeneration and not for dentin. In terms of physical structure and chemical composition, dentin is similar to bone. The ultimate constituents of teeth and bone are identical elements: collagen, HA, and non-collagen proteins [25]. In our study, we hypothesize that the use of nanofibrous scaffolds with a high surface area and interconnected porous characteristics is highly suited for dentin mineralization.

In this study, Asp-loaded nanofibrous scaffolds (PCL/nHA/Asp) was fabricated by electrospinning. SEM of the scaffold (PCL/nHA/Asp) and the control scaffolds (PCL, PCL/nHA) were performed immediately after fabrication. SEM images of the scaffolds confirmed the formation of nanofibers, which can serve as an ECM. Nanofiber formation is essential since it can support interfibrillar and intrafibrillar apatite nucleation and growth of crystals.

Calcium phosphate ceramics (or bioceramics) have gained extensive use in bone regeneration, which is attributed to their properties of osteoconduction and osteoinduction [20,26]. Among bioceramics, nHA has been extensively studied in the field of hard tissue engineering due to its close resemblance in chemical and structural aspects to biomineralized structures. It offers biocompatibility and bioactivity, causing no inflammatory responses. Additionally, it possesses advantageous characteristics such as a slow degradation rate, the ability to adsorb proteins, and the controlled release of calcium and phosphate ions. These attributes play a role in regulating cell tissue biomineralization. Nevertheless, the fragility, tendency to dissolve, and challenges in shaping isolated nHA hinder its practical use as a temporary substitute at injured sites [27]. Hence, the proposal of integrating nHA into PCL scaffolds has emerged as a solution for bone regeneration. Similarly, in our study, nHA was incorporated into PCL for dental mineralization. EDX was taken initially to confirm the presence of nHA in the scaffold. EDX showed the presence of calcium and phosphate, which confirms the presence of nHA in the scaffold. The infrared spectra of the amino acid residues have been acquired in 1,800-1,400 cm^-1^ region [28]. It has been established that amino acid residues of arginine, asparagine, glutamine, Asp and glutamic acids, lysine, tyrosine, histidine, and phenylalanine have intensive absorption in this spectral region [28]. FTIR analysis showed characteristic functional groups corresponding to carbonyl stretching of PCL, OH peak of nHA, and NH2 peaks corresponding to Asp. X-ray diffraction analysis showed the characteristic peak corresponding to the individual component and also showed crystalline peaks for the Asp.

The mineralization study was conducted by immersing the scaffold in SBF and characterized using SEM and EDX. SEM serves as a qualitative analytical method used to observe and analyze surface characteristics and structures at a microscopic level. After 7 days of incubation, in PCL scaffold, tiny globular-shaped crystals were observed on the surface of the fiber and not within the pore network. PCL/nHA scaffold showed dense deposits in between the nanofibers. In PCL/nHA/Asp scaffold, increase in the width of the fibers was observed due to the uniform deposition of minerals along the fibers. Small spherical seeding crystals were observed along the fibers, serving as the initial points for crystal growth to occur. After 14 days of mineralization in SBF, PCL/nHA/Asp scaffold showed cauliflower-like shaped crystals, resembling crystals of HA. In both PCL and PCL/nHA scaffolds, the crystals were randomly distributed, and mineralization was observed only on the surface and not within the pore network for 14 and 21 days. After 21 days of mineralization, PCL/nHA/Asp showed crystal nucleation and growth, both on the surface and within the fibers (pore structure). SEM images showed the distribution of crystal formation all over the fibers. Crystal growth was seen along a uniform axis, producing uniform deposits all over the fibers. Increasing the incubation time in SBF from 14 days to 21 days led to substantial crystal nucleation and growth. The elemental analysis confirmed the augmentation in the crystal deposits on the scaffolds overtime during the incubation period. EDX confirmed the gradual rise in the crystal deposits on the PCL/nHA and PCL/nHA/Asp scaffolds as the incubation time progressed. Higher calcium and phosphate levels were observed within 14 days on PCL/nHA/Asp. The negatively charged Asp may have contributed to the enhanced mineral deposition due to its affinity for calcium that was observed at the 14-day analysis. Moreover, it continued to remain stable at the 21-day analysis. This could potentially reduce the contact time required to support dental mineralization. Though PCL/nHA continued to increase the mineral deposit at 21 days, SEM analysis revealed unevenly distributed intensive mineral deposits. At 14 and 21 days, PCL did not significantly increase the mineral deposit, validating the importance of Asp and HA in mineralization.

Our study demonstrated that PCL/nHA/Asp electrospun scaﬀold is a potential bioactive material. Its scope in dental mineralization as well as bone tissue regeneration applications by providing a conducive environment for mineral deposition must be further explored. It can potentially provide mechanical support to weakened dentin, enhancing its strength and resilience against masticatory forces. The scaffold can be tailored to fit various cavity shapes and sizes, ensuring optimal adaptation and efficacy in different clinical scenarios.

﻿﻿﻿One of the study's limitations is that the scaffold's mechanical properties were not evaluated. The PCL matrix's compressive strength may have been affected by the addition of Asp. This has bearing on how the scaffold is used at the base of a restoration. Studies on cytotoxicity and cell viability are necessary to confirm the material's biocompatibility. It is clear that in vivo research is necessary to validate the feasibility of developing a fibrous scaffold appropriate for the prospective uses described in this article.

## Conclusions

In this study, Asp-incorporated nanofibrous scaffolds were fabricated through the electrospinning technique successfully. The electrospun nanoﬁbers were developed by incorporating PCL, nHA, and Asp. The nanoﬁbers were bead-free smooth, randomly oriented, and loaded with Asp. The EDX spectra of PCL/nHA/Asp composite nanofibrous scaffold revealed broad peaks and corresponded to the amorphous form, while the sharp peaks corresponded to the specific crystalline structure of nHA. FTIR analysis showed specific functional groups corresponding to PCL, nHA, and Asp. The scaﬀolds incorporated with Asp exhibited higher mineralization potential with an apatite-like crystal formation. The PCL/nHA/Asp scaffold exhibited an enhanced ability to stimulate the initial nucleation of apatite and the subsequent growth of calcium phosphate crystals in vitro, as opposed to PCL-only fibers. The specific apatite species that initiated on the PCL/nHA/Asp scaffolds exhibited typical HA characteristics after 14 and 21 days supporting the evidence of mineralization.
